# Case report: Late-onset hypertrophic pyloric stenosis in a 3-year-old boy: It is never too late

**DOI:** 10.3389/fped.2022.949144

**Published:** 2022-08-16

**Authors:** Onofrio Iacoviello, Giuseppe Verriello, Stefania Castellaneta, Stefano Palladino, Michela Wong, Girolamo Mattioli, Paola Giordano, Ruggiero Francavilla, Fernanda Cristofori

**Affiliations:** ^1^Department of Pediatrics, Ospedale Pediatrico “Giovanni XXIII,” University of Bari, Bari, Italy; ^2^Department of Radiology, Ospedale Pediatrico “Giovanni XXIII,” University of Bari, Bari, Italy; ^3^Department of Pediatric Surgery, Istituto “Giannina Gaslini,” University of Genoa, Genoa, Italy

**Keywords:** pyloromyotomy, laparoscopy, hypertrophic pyloric stenosis, vomit, surgery

## Abstract

Hypertrophic Pyloric Stenosis (HPS) represents a relatively rare occurrence beyond infancy. Here, we present the case of a barely 3-year-old boy diagnosed with late-onset HPS and successfully treated with extra-mucosal pyloromyotomy. We review the literature, challenging the principle that more aggressive surgical approaches should be preferred over less invasive ones.

## Introduction

Hypertrophic pyloric stenosis (HPS) represents the most common cause of gastric outlet obstruction (GOO) in infants, being a rare occurrence beyond infancy.

Here, we present the case of a barely 3-year-old child, who was admitted to the Emergency Department with symptoms of persistent non-bilious vomiting over the past 15 days. Abdominal ultrasound demonstrated thickening of the pylorus, while an upper GI series revealed obstruction to gastric emptying.

Endoscopic balloon dilation (EBD), despite providing symptomatic improvement, failed to normalize gastric emptying. He was treated with extra mucosal pyloromyotomy, the gold standard of treatment in infantile forms, with a good outcome and fast recovery.

After reviewing the literature, we found that conservative pyloric surgery, which represents the gold standard treatment for infantile forms, is also effective and safe in late-onset forms of HPS and should be preferred over more invasive surgical approaches whenever possible.

## Case description

A 2-year-and-11-month-old male was admitted to the emergency department for persistent non-bilious vomiting episodes, which had started 15 days before, and became more frequent over time. Episodes typically occurred 4–5 h after his meals and were preceded by hypersalivation and rumination. A weight loss of 3.5 kg had occurred over the last 4 weeks, while bowel function had remained regular. One month before, he had suffered from a self-limited upper respiratory tract infection. Past medical and family history was otherwise unremarkable.

**FIGURE 1:**
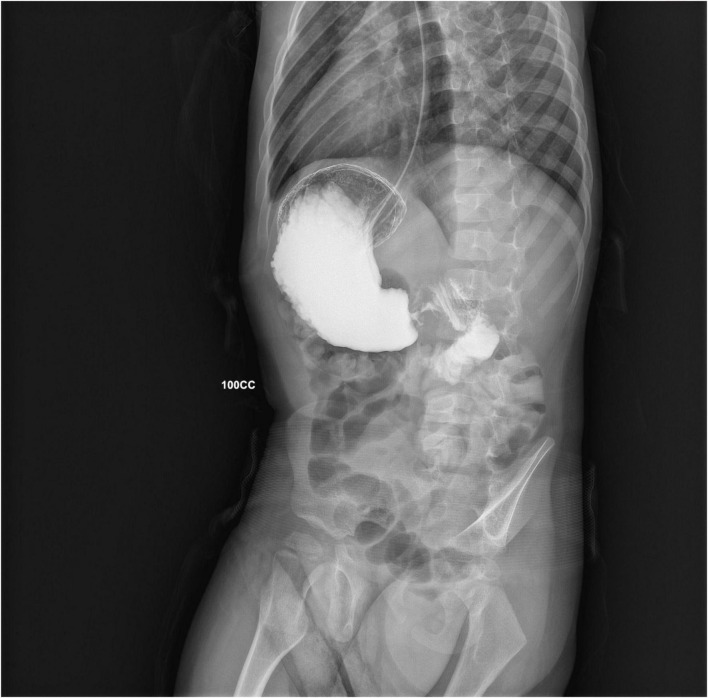
A right anterior oblique projection from the GI series, showing accumulation of contrast medium into a dilated antrum (*shoulder sign*) and a narrow string-like pyloric channel (*string sign*).

**FIGURE 2:**
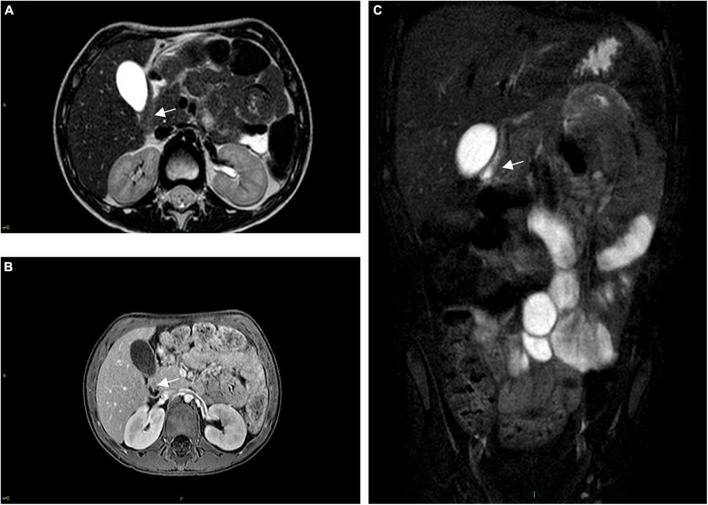
Pre-operative abdominal MRI. **(A)** Axial TSE-T2w, **(B)** axial GRE-T1w, and **(C)** coronal turbo-spin echo T2w FAT-SAT sequences are shown, all demonstrating a thickened and narrowed pylorus (arrow), without a visible lumen.

On admission, physical examination was normal, with a soft, symmetric, non-tender abdomen with no palpable masses, with no signs of significant dehydration. Arterial Blood Gas analysis showed signs of mild metabolic alkalosis (pH 7.49, HCO3 28 mmol/L). Within 2 h of being admitted to the hospital, however, the patient presented some projectile, non-bilious vomiting. An abdominal X-ray showed significant gastric distension, with no signs of pneumoperitoneum, while an abdominal ultrasound (US) showed a thickened pylorus with a single-wall thickness of 7 mm and a length of 16 mm. An upper GI series (Figure 1) revealed an accumulation of contrast medium in the dilated gastric antrum (*shoulder sign*), a narrow string-like pyloric channel (*string sign*), and delayed gastric emptying, with only a modest amount of contrast medium passing through pylorus over the next 60 min. An urgent CT scan was performed to exclude extrinsic causes of gastric outlet obstruction (GOO), showing an elongated pyloric canal (approximately 20 mm) without a visible lumen and a thickened wall (single wall thickness of 6 mm). These measurements were later confirmed by an abdominal MRI (Figure 2).

A nasogastric tube was placed, and the patient was started on parenteral nutrition. Upper endoscopy was performed 48 h later, showing distal esophagitis with no evidence of gastric ulcers, mucosal webs, or foreign bodies. Endoscopic balloon dilation (EBD) was attempted, and biopsies were performed, testing negative for *Helicobacter pylori* infection and showing no inflammatory infiltrate. The patient was started on PPIs and alginate, and 48 h after the procedure, a normal-caloric liquid formula was administered with good tolerance.

Despite significant clinical improvement, with no more episodes of vomiting reported, the post-operative GI series performed on the 5th post-operative day still showed delayed gastric emptying (complete emptying requiring approximately 4 h) and the persistence of a narrowed pyloric outlet. The patient was, therefore, referred to surgery. A laparoscopic extra mucosal pyloromyotomy was performed with prompt normalization of transpyloric transit time (as demonstrated by a barium swallow) and good tolerance to refeeding 24 h postoperatively.

Over the subsequent 6 months, the patient has shown good clinical conditions with no more episodes of vomiting, or other relevant symptoms reported during follow-up evaluations. Weight loss was completely recovered over the first 30 days from hospital discharge.

## Discussion

Gastric outlet obstruction refers to a group of heterogeneous conditions that prevent or delay the passage of gastric content into the duodenum (1). It occurs in approximately 2–5 per 1,000 infants and children per year (2). Non-bilious vomiting, dehydration, failure to thrive, and weight loss are the most common clinical manifestations.

Hypertrophic pyloric stenosis is, by far, the most common cause of gastric outlet obstruction (GOO) in young infants, with an incidence of 1–4 in 1,000 live births (3). It is defined as hyperplasia of the smooth muscle fibers of the pylorus, causing a narrowing of its lumen. HPS usually appears within the first 2–8 weeks of life (4). More than 90% of HPS cases occur between 3 and 10 weeks of age, being instead a rare finding after 12 weeks (4, 5). Males are more commonly affected with a male-to-female ratio of 4:1 (6). Its etiopathogenesis is almost invariably idiopathic, with genetic, hormonal, and environmental factors all playing a role.

When HPS is excluded, the incidence of GOO falls dramatically (1 in 100,000 births by some estimates (7)). These non-hypertrophic, least common causes of GOO can be classified into three groups: *congenital* (esophageal aplasia, atresia, diaphragms, and webs; luminal obstructions); *secondary* (to acid peptic diseases, neoplasm, chemical injury, or foreign body ingestion); or *primary acquired* (Jodhpur disease), characterized by defects in pyloric motility with no hypertrophy (7–9).

HPS beyond infancy is a rare occurrence (7, 10, 11). Its etiology in older patients is controversial. Most authors, however, seem to agree that late-onset HPS is due to the persistence of the infantile form, which becomes clinically evident only at a later stage, when a triggering event, such as inflammation, edema, or spasms, precipitates pyloric occlusion (12, 13).

If GOO is suspected, an early patient assessment should include blood gas analysis, electrolytes, and fluid resuscitation; placement of a nasogastric tube allows for prompt gastric decompression. Abdominal ultrasound is the first-line examination (14), allowing the identification of a thickened pylorus (transverse section > 3 mm and length > 12 mm (15, 16)). An upper GI series allows to rule out other potential causes of projectile vomiting, such as malrotations and severe gastroesophageal reflux (14). In HPS, the pyloric lumen appears narrowed, with evidence of a string-like passage between the antrum and the duodenum (string sign), occasionally with a duplicated appearance due to puckering of the mucosa (double-track sign) (17). MRI and CT scans prove helpful to rule out extrinsic causes of obstruction. Total parenteral nutrition is indicated and should be initiated upon diagnosis confirmation, while PPIs may be of help in patients with persistent episodes of vomiting to prevent esophagitis (18). Pyloromyotomy has become the standard of care for infantile HPS; postoperatively, feeding can be resumed within 6 h, and the patient is discharged within 2 days. Laparoscopy is rapidly gaining ground over the open approach, although no systematic review has yet shown a clear advantage of one over the other, neither in terms of efficacy nor when it comes to post-operative complications (19).

When it comes to late-onset HPS, the data in the literature are conflicting, and several different procedures have been reported (20) (Table 1).

**TABLE 1:** Published case reports and one case series (Boybeyi et al.) (18) of late-onset HPS.

Paper, year	Cases reported	Age	Treatment	Comment
Selzer et al. (29)	1	14 year	HMP	–
Mahalik et al. (14)	1	4,5 years	OP	–
Boybeyi et al. (18)	11	3,6 years (mean age)	- EBD (4 pts) - EBD + B-I (1 pt) - B-I (6 pts)	- Concerns over mucosal lacerations and late diverticula; - Concerns over abnormality in pyloric motility.
Bajpai et al. (30)	1	8 years	HMP + distal gastrectomy	ns
Parnall et al. (31)	1	15 years	- Botulinum toxin injection (failure) - B-I	- Presence of a gastric pseudo-diverticulum and gastric mucosal scarring
Wolf et al. (32)	1	17 years	LP	–
Al-Mayoof and Doghan (33)	1	7 months	LP	–
Bartlett et al. (34)	1	12 years	HMP	–
Oswari et al. (35)	1	11 years	B-I	ns
Skafi et al. (36)	1	4 months	LP	–
Plessi et al. (2)	1	12 years	- EBD + electrosurgical incisions (failure) - Distal gastrectomy Roux-en-y anastomosis	- Severity of obstruction - Failure of previous treatment

For each case, the treatment of choice is indicated. The “Comment” column indicates, when specified by the authors, the reason why a demolitive surgery was preferred over pyloromyotomy or pyloroplasty. OP, Open pyloromyotomy; LP, Laparoscopic pyloromyotomy; HMP, Heineke-Mikulicz pyloroplasty; EBD, Endoscopic Balloon Dilation; B-I, Billroth I; ns, Reason not specified; pt, patient.

Boybeyi et al. suggested a possible treatment algorithm, indicating gastrectomy followed by Billroth I reconstruction whenever two consecutive EBDs attempts have failed or in those circumstances in which EBD cannot be performed (18). The rationale of gastrectomy, as compared to less invasive approaches, is related to possible complications, such as mucosal lacerations, pyloric scarring, diverticula formation, abnormal post-operative pyloric motility, and function (13, 18, 21–23). However, most of these concerns appear to be based on anecdotal evidence and are not corroborated by recent data (24–27).

Other authors underline some other limitations of less invasive procedures, such as the inability to visualize the gastric and duodenal mucosa, or the impossibility to obtain large histologic specimens (23, 26), which should be, however, evaluated on a case-to-case basis.

Some studies reporting on gastric emptying after conservative pyloric surgery seem to agree that conservative pyloric surgery produces no alterations in gastric emptying or intragastric distribution of meals and does not increase the risk of complications, such as gastric and duodenal ulcerations (27, 28).

In our case, gastric emptying did not improve significantly after EBD but underwent complete normalization after pyloromyotomy, with no return of symptoms after 6 months from the procedure.

The case report herein presented shows that a high degree of suspicion for HPS should be maintained even beyond infancy, to promptly identify and treat it.

Endoscopic balloon dilation represents a relatively safe, minimally invasive first-line approach in late-onset forms of HPS. As to the surgical approach to be adopted in refractory forms, the evidence is still insufficient. Pyloromyotomy is the gold standard treatment in infants. However, its efficacy and possible complications in older patients are yet to be established. The adoption of more invasive strategies, including distal gastrectomy and subsequent Billroth I reconstruction, should be decided on an individual basis and should be reserved for those cases, in which: (a) there are doubts on adequate pyloric motility and functionality (e.g., Jodhpur disease, scarring); (b) pyloromyotomy/pyloroplasty is made technically difficult by anatomy or by the thickness of the pylorus; (c) full-thickness biopsies are required to exclude other diseases, such as neoplasms; or (d) more conservative approaches have failed, or relapses have occurred.

## Data availability statement

The original contributions presented in this study are included in the article/supplementary material, further inquiries can be directed to the corresponding author.

## Ethics statement

Ethical review and approval was not required for the study on human participants in accordance with the local legislation and institutional requirements. Written informed consent was obtained from the minor(s)’ legal guardian/next of kin for the publication of any potentially identifiable images or data included in this article.

## Author contributions

OI and GV drafted the initial manuscript, and reviewed and revised the manuscript. GM and MW carried out the surgical procedure and critically reviewed the manuscript, providing important insights concerning the surgical aspects of the cases. SC and FC collected the data, conducted the endoscopic study, and reviewed and revised the manuscript. RF and PG contributed to the conception and design of the report and wrote sections of the manuscript. SP conducted the radiological studies, selected the images, drafted their description, and critically reviewed the manuscript. All authors approved the final manuscript as submitted and agreed to be accountable for all aspects of the work.
